# Cardiometabolic Multimorbidity Associated with Moderate and Severe Disabilities: Results from the Study on Global AGEing and Adult Health (SAGE) Wave 2 in Ghana and South Africa

**DOI:** 10.5334/gh.1188

**Published:** 2023-03-01

**Authors:** Peter Otieno, Gershim Asiki, Justice Moses K. Aheto, Calistus Wilunda, Richard E. Sanya, Welcome Wami, Daniel Mwanga, Charles Agyemang

**Affiliations:** 1 African Population and Health Research Center, Nairobi, Kenya, NL; 2 Department of Public & Occupational Health, Amsterdam UMC, University of Amsterdam, Amsterdam Public Health Research Institute, Amsterdam, NL; 3 Amsterdam Institute for Global Health and Development, Amsterdam, NL; 4 African Population and Health Research Center, Nairobi, Kenya, SE; 5 Department of Women’s and Children’s Health, Karolinska Institutet, Stockholm, SE; 6 Department of Biostatistics, School of Public Health, College of Health Sciences, University of Ghana, GH; 7 University of Amsterdam, Department of Global Health, Paasheuvelweg 25, Amsterdam, NL

**Keywords:** Cardiometabolic diseases, multimorbidity, disability, latent class analysis

## Abstract

**Background:**

Integrated management of cardiometabolic diseases is crucial in improving the quality of life of older persons. The objective of the study was to identify clusters of cardiometabolic multimorbidity associated with moderate and severe disabilities in Ghana and South Africa.

**Methods:**

Data were from the World Health Organization (WHO) study on global AGEing and adult health (SAGE) Wave-2 (2015) conducted in Ghana and South Africa. We analysed the clustering of cardiometabolic diseases including angina, stroke, diabetes, obesity, and hypertension with unrelated conditions such as asthma, chronic lung disease, arthritis, cataracts, and depression. The WHO Disability Assessment Instrument version 2.0 was used to assess functional disability. We used latent class analysis to calculate the multimorbidity classes and disability severity levels. Ordinal logistic regression was used to identify the clusters of multimorbidity associated with moderate and severe disabilities.

**Results:**

Data from 4,190 adults aged over 50 years were analysed. The prevalence of moderate and severe disabilities was 27.0% and 8.9% respectively. Four latent classes of multimorbidity were identified. These included a relatively healthy group with minimal cardiometabolic multimorbidity (63.5%), general and abdominal obesity (20.5%), hypertension, abdominal obesity, diabetes, cataracts, and arthritis (10.0%), and angina, chronic lung disease, asthma, and depression (6.0%). Compared to the participants with minimal cardiometabolic multimorbidity, the odds of moderate and severe disabilities were higher among participants with multimorbidity comprising hypertension, abdominal obesity, diabetes, cataract and arthritis [aOR = 3.0; 95% CI 1.6 to 5.6], and those with angina, chronic lung disease, asthma and depression [aOR = 2.7; 95% CI 1.6 to 4.5].

**Conclusions:**

Cardiometabolic diseases among older persons in Ghana and South Africa cluster in distinct multimorbidity patterns that are significant predictors of functional disabilities. This evidence may be useful for defining disability prevention strategies and long-term care for older persons living with or at risk of cardiometabolic multimorbidity in sub-Saharan Africa.

## Background

Cardiometabolic multimorbidity, defined as having two or more coexisting cardiometabolic diseases (CMDs), is a major global health challenge to healthcare systems [1,2]. The findings of the 1990–2019 Global Burden of Diseases show that people are living longer, but with more chronic diseases and disabilities [3]. The number of people living with CMDs, such as coronary artery disease, stroke, hypertension, and diabetes has been rising globally [4,5]. Importantly, CMDs often coexist with obesity and insulin resistance [6]. Characterizing these disease states reveals that discordant multimorbidity, the simultaneous occurrence of other diseases with unrelated pathophysiology or pharmacological treatments, such as chronic lung diseases and musculoskeletal disorders, are common among people with CMDs [7,8,9].

The population of older persons in sub-Saharan Africa (SSA) is projected to triple to 235.1 million in 2050 from 74.4 million in 2020 [10]. Ghana and South Africa are at different stages of epidemiological and demographic transitions, but are experiencing a rapid increase in the proportion of older persons [11]. Recent studies show a rising prevalence of cardiometabolic multimorbidity in Ghana and South Africa [12,13,14,15]. With this rapid demographic transition, active ageing has become a priority for long-term healthcare policies in SSA [16,17]. The main goal of active ageing is to maintain the capacity to perform the activities of daily living (ADL) across the life course [18]. Thus, measuring disability severity levels is crucial in understanding the consequences of ageing and planning long-term care programs. Disability is a complex process that transcends physical limitations [19]. The International Classification of Functioning Disability and Health defines disability as a decline in three levels of functioning: bodily, person, and societal [20]. Hence, disability comprises impairment in bodily functions, limitations in ADLs, and restrictions on societal participation [18].

Although recent studies have attributed functional decline to the presence of multimorbidity, several research gaps still exist [1,21,22,23,24,25]. First, the majority of the existing literature is based on disease counts or indices with inadequate information on specific concordant or discordant multimorbidity clusters to guide clinical or social interventions [26,27]. Second, a lack of homogeneity in the conceptualisation of multimorbidity has hindered the comparison of findings [28]. Third, the majority of studies have estimated the prevalence of disability using a binary classification approach [29,30,31,32]. Such an approach may overlook the heterogeneity of the disability severity levels frequently observed among the older population.

Most literature on multimorbidity is available in high-income countries, where disease burdens and healthcare systems differ from those in SSA [33], hence the need for similar research in SSA. Consequently, the WHO Study on global AGEing and adult health (SAGE) project was designed to address the emerging evidence gaps on ageing and well-being in low and middle-income countries [19]. In 2020, one analysis from the SAGE Wave 1 study revealed that the prevalence of severe disability was three to five times higher in South Africa and Ghana than in China and Mexico [34]. Whilst the findings of the SAGE study show high levels of severe disability among older adults in Ghana and South Africa, a more comprehensive unpacking of specific multimorbidity clusters may provide opportunities for the integrated management of disease combinations with adverse impacts on functional health. Using the 2015 WHO SAGE Wave 2 survey in Ghana and South Africa, the objective of the current study was to determine the concordant and discordant cardiometabolic multimorbidity combinations and explore the associations with levels of disability (i.e. no disability, moderate, and severe).

## Methods

### Study design

Data were from the WHO SAGE Wave 2 survey conducted in Ghana and South Africa in 2015 [35]. The study design has been previously published [19]. The SAGE survey is an ongoing population-based longitudinal study that aims to provide reliable evidence on ageing and well-being from nationally representative samples of persons aged 50 years and older [11]. The primary sampling units were stratified across urban and rural areas in each country to capture socioeconomic differences and lifestyle behaviours. A standard protocol for the WHO SAGE survey was used in all the study countries [19]. Briefly, the study participants were selected using a two-stage stratified sampling design. Stage 1 comprised of a selection of clusters based on probability, proportional to the number of households in the cluster. Households in each cluster were listed in stage 2 and a simple random sample was drawn from the listing frame. Eligible participants comprised all listed household members aged 50 years and older residing in the sampled households.

### Data sources

Data used in the current study were collected using interviewer-administered structured questionnaires modified from the World Health Survey tool [35]. Detailed information on the study tools and data collection procedures is provided elsewhere [19]. The current analysis focused on the screening outcomes for chronic conditions and functional disability. The chronic conditions comprised CMDs, such as angina pectoris, stroke, diabetes, hypertension, obesity, and conditions such as arthritis, asthma, chronic lung disease, depression, and cataracts. Other variables included socio-demographic information such as age, sex, place of residence (rural and urban), and employment.

#### Measurement of variables

The screening for chronic diseases was based on self-reported history of clinical diagnosis, algorithms for symptomatology, physical measurements, and anthropometrics. We extracted the self-reported history of clinical diagnosis of angina pectoris, stroke, diabetes mellitus, hypertension, arthritis, asthma, chronic lung disease, depression, and cataracts. The diagnosis was ascertained using the screening question: ‘Has a healthcare professional ever told you that you have (disease name)?’ In addition, based on the information available, WHO-recommended symptomatology algorithms were used to screen for angina pectoris, arthritis, asthma, chronic lung disease, and depression [36,37,38]. Details of the symptomatology algorithms are shown in the online supplementary file 1.

The physical measurements comprised screening for systolic blood pressure (SBP), diastolic blood pressure (DBP), and anthropometrics including waist circumference (cm), weight (kg), and height (m). Screening blood pressure was recorded as the average of the last two BP readings. Hypertension was defined as SBP ≥ 140 mmHg and/or DBP ≥ 90 mmHg or the previous diagnosis of hypertension by a professional health care provider or being on hypertensive therapy [39]. Abdominal obesity was defined using WHO guidelines as waist circumference ≥94 cm for men, or ≥80 cm for women [40]. General obesity was defined as body mass index ≥30.0 kg/m^2^ [41]. Participants with a history of clinical diagnosis or treatment for any of the chronic conditions but screened negative based on the symptomatology algorithms or physical measurements were considered to have the condition.

#### Inclusion and exclusion criteria

The original sample of participants aged 50 years and older surveyed in the two study countries was 5,757 (Ghana: n = 3,575 and South Africa: n = 2,182). Participants were included in the current analysis if they had valid data on the key variables: disability status, chronic diseases such as angina pectoris, stroke, diabetes mellitus, hypertension, obesity, arthritis, asthma, chronic lung disease, depression, and cataracts and sociodemographic characteristics comprising sex, age, and employment. Participants (n = 1, 529) for which data on key variables were not captured or judged as invalid were excluded. Since the causes of missing information were not ascertained, we did not apply missing data techniques to avoid further uncertainty in the imputation models. Thus, the final analysis included 4,190 participants.

Given the exclusion of participants with missing data on the key study variables, the characteristics of the study participants with complete data were compared to those with incomplete data and no differences were found based on age and sex (see online supplementary file 2).

### Definition of variables

#### Outcome variable

Disability status was the main outcome variable. The WHO Disability Assessment Instrument version 2.0 (WHODAS 2.0) was used to screen for disability. The WHODAS 2.0 is a cross-culturally validated disability assessment tool comprising six domains assessed using a 12-item scale with two items per domain [42]. The domains comprise self-care, cognition and communication, mobility, life activities, interpersonal relations, and participation. The global score is the sum of the 12 items from the six domains expressed on a continuous scale ranging from 0 (no disability) to 100 (full disability) [42].

#### Explanatory variables

The main explanatory variable was cardiometabolic multimorbidity, defined as having two or more concordant or discordant cardiometabolic multimorbidities. Concordant cardiometabolic multimorbidity was defined as the simultaneous presence of two or more CMDs including angina pectoris, stroke, diabetes, hypertension, and obesity [43]. Discordant cardiometabolic multimorbidity was defined as the simultaneous presence of at least one of the CMDs and one or more chronic diseases with unrelated pathophysiology or pharmacological treatment plans such as chronic lung disease, arthritis, cataracts, and depression [44]. We computed clusters of concordant cardiometabolic multimorbidities comprising angina pectoris, stroke, diabetes, hypertension, obesity, and discordant multimorbidity including chronic lung diseases, arthritis, cataracts, and depression. Other explanatory variables comprised sociodemographic factors such as age, sex, marital status, education level, employment status, and place of residence (urban or rural).

### Data Analysis

We used descriptive statistics comprising frequencies, means, and standard deviations to summarise the characteristics of the study participants and disability patterns in a pooled dataset of the study countries, while adjusting for survey weights.

#### Latent class analysis

We used latent class analysis (LCA) to identify distinct groups of multimorbidity classes and disability levels. The LCA is a methodological approach used to identify groups of participants with homogenous response patterns to a set of observed variables [45]. We determined the optimal number of latent classes using the adjusted Bayesian Information Criterion (aBIC) and the consistent Akaike Information Criterion (CAIC). The aBIC and CAIC have been previously used as robust indicators for determining the optimal number of classes for latent variables [46,47]. First, the aBIC and CAIC were used to compare several plausible models. The models with the lowest values of aBIC and CAIC were finally selected as the best fitting models [48,49]. The posterior probabilities were used to determine the likelihood of multimorbidity class membership and levels of disability. Finally, the participants were grouped into the cardiometabolic multimorbidity classes and disability levels with the highest-class probability.

#### Hierarchical cluster analysis

We conducted a supplementary analysis of the multimorbidity patterns using the agglomerative hierarchical cluster analysis with the average linkage method (HCA) [50]. First, a proximity index was used to group the individual chronic diseases into a single cluster. Next, the chronic disease clusters were gradually merged with the most closely related clusters until a single cluster with all the elements was obtained. We used a dendrogram plot and Jaccard similarity coefficient to assess the cardiometabolic multimorbidity patterns [51].

#### Ordinal regression

Given the ordinal nature of the disability severity levels (i.e. no disability, moderate, and severe) identified from the LCA, weighted ordinal regression was used to model the association of cardiometabolic multimorbidity classes with disability levels on a pooled dataset from the two study countries. Because of the clustered design of the sample, robust variance estimates (Huber-White sandwich estimator) were used for the correction of standard errors to adjust for the correlation among responses within the same household [52].

Bivariable ordered logistic regression analysis with levels of disability as the outcome variable was first fitted for each of the multimorbidity classes, followed by a multivariable model adjusting for socio-demographic characteristics namely age, sex, education, employment status, and place of residence. There was no evidence of a violation of the assumption of parallel slopes using the command ‘*brant*’ in Stata 17.0 (StataCorp LP, Texas, USA). The likelihood ratio test was used to compare the goodness of fit of the models. We used the adjusted odds ratio (aOR) and 95% Confident Interval (CI) to interpret the strength and direction of associations.

All statistical analyses were carried out using Stata 17.0 (StataCorp LP, Texas, USA) and accounted for the complex sampling design used in the SAGE survey.

## Results

### Characteristics of participants

The socio-demographic and health characteristics of the study participants are presented in Table 1. A total of 4,190 participants were included in the analysis. The mean age was 61.6 years. In general, most of the participants were women (54%), had a primary (35.7%) or secondary level of education (32.2%), were self-employed (33.3%), and lived in urban areas (70.9%). The most prevalent CMDs were abdominal obesity (56.4%) and hypertension (48.8%). Concordant and discordant cardiometabolic multimorbidities were observed in 45.9% and 33.8% of the participants respectively. Varying patterns in the distribution of multimorbidity were observed between the two study countries. The highest prevalence of concordant and discordant cardiometabolic multimorbidity was observed in South Africa (56.3% and 38.4% respectively).

**Table 1:** Sociodemographic and health characteristics of the study participants.


	COUNTRY		POOLED DATA
	
CHARACTERISTICS (%)	GHANA, N = 3,128	SOUTH AFRICA, N = 1,062	BOTH COUNTRIES, N = 4,190

Age, (mean) SD	61.9 (9.7)	61.4 (8.4)	61.6 (8.9)

Sex			

Male	47.7	44.9	46.0

Female	52.3	55.1	54.0

Education			

No formal education	41.5	17.0	26.7

Primary	28.3	40.5	35.7

Secondary	26.5	35.8	32.2

Tertiary	3.7	6.6	5.5

Employment			

Public	7.8	9.8	9.0

Private	4.4	43.5	28.0

Self-employed	69.7	9.6	33.3

Informal employment	16.2	29.0	24.0

Unemployed	1.9	8.2	5.7

Place of residence			

Urban	47.9	85.8	70.9

Rural	52.1	14.2	29.1

Chronic diseases			

Abdominal obesity	47.0	62.5	56.4

Hypertension	37.1	56.4	48.8

General obesity	13.4	39.0	28.9

Arthritis	20.4	21.4	21.0

Asthma	8.3	16.3	13.2

Cataract	7.2	9.5	8.6

Diabetes	2.6	12.0	8.3

Angina	8.4	6.0	6.9

Chronic lung disease	4.5	7.4	6.2

Depression	4.5	6.4	5.6

Stroke	1.2	2.4	1.9

^†^ Concordant cardiometabolic multimorbidity	29.9	56.3	45.9

^‡^ Discordant cardiometabolic multimorbidity	26.6	38.4	33.8


Cells are weighted row percentages unless otherwise specified. ^†^ Concordant cardiometabolic multimorbidity is defined as the simultaneous presence of two or more cardiometabolic diseases including angina pectoris, stroke, diabetes, hypertension, and obesity. ^‡^ Discordant cardiometabolic multimorbidity is defined as the simultaneous presence of at least one cardiometabolic disease and one or more chronic diseases with unrelated pathophysiology or pharmacological treatment plans such as chronic lung disease, asthma, arthritis, cataracts, and depression.

### Findings of latent class analysis

#### Cardiometabolic multimorbidity classes

**Figure 1:**
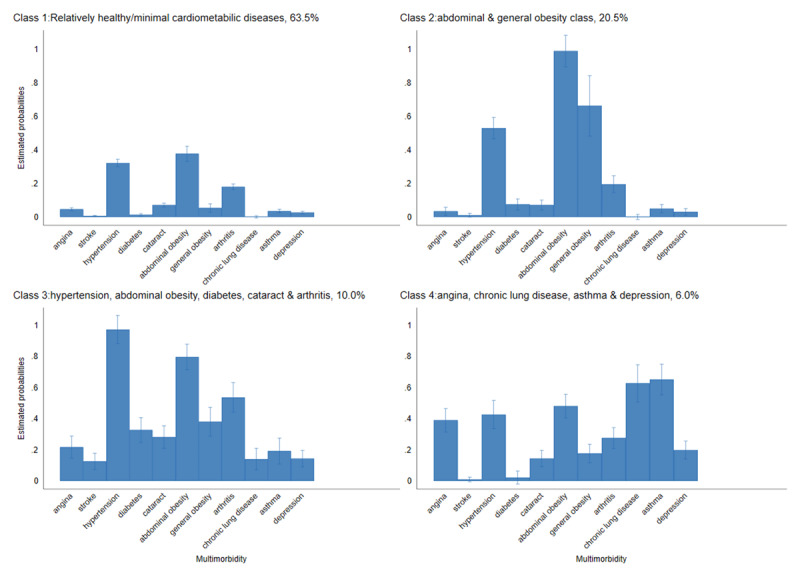
Latent classes of concordant and discordant cardiometabolic multimorbidity.

The multimorbidity classes are shown in Figure 1. We compared LCA models with two to five classes (online supplementary file 3). Although the five-class model had a slightly lower AIC than the four-class (AIC_5class_ = 31748.2 vs. AIC_4class_ = 31791.5), further inspection showed that the four-class model exhibited clearer separation between latent classes and had the lowest aBIC. Thus the four-class model was finally selected. Class one (interpreted as the ‘relatively healthy group’) comprised participants with minimal cardiometabolic multimorbidity (63.5%). Class two comprised participants with high probabilities of general and abdominal obesity (20.5%). Class three comprised participants with high probabilities of multimorbidity of hypertension, abdominal obesity, diabetes, cataracts, and arthritis (10.0%). Class four comprised participants with high probabilities of multimorbidity of angina, chronic lung disease, asthma, and depression (6.0%).

#### Disability levels

**Figure 2:**
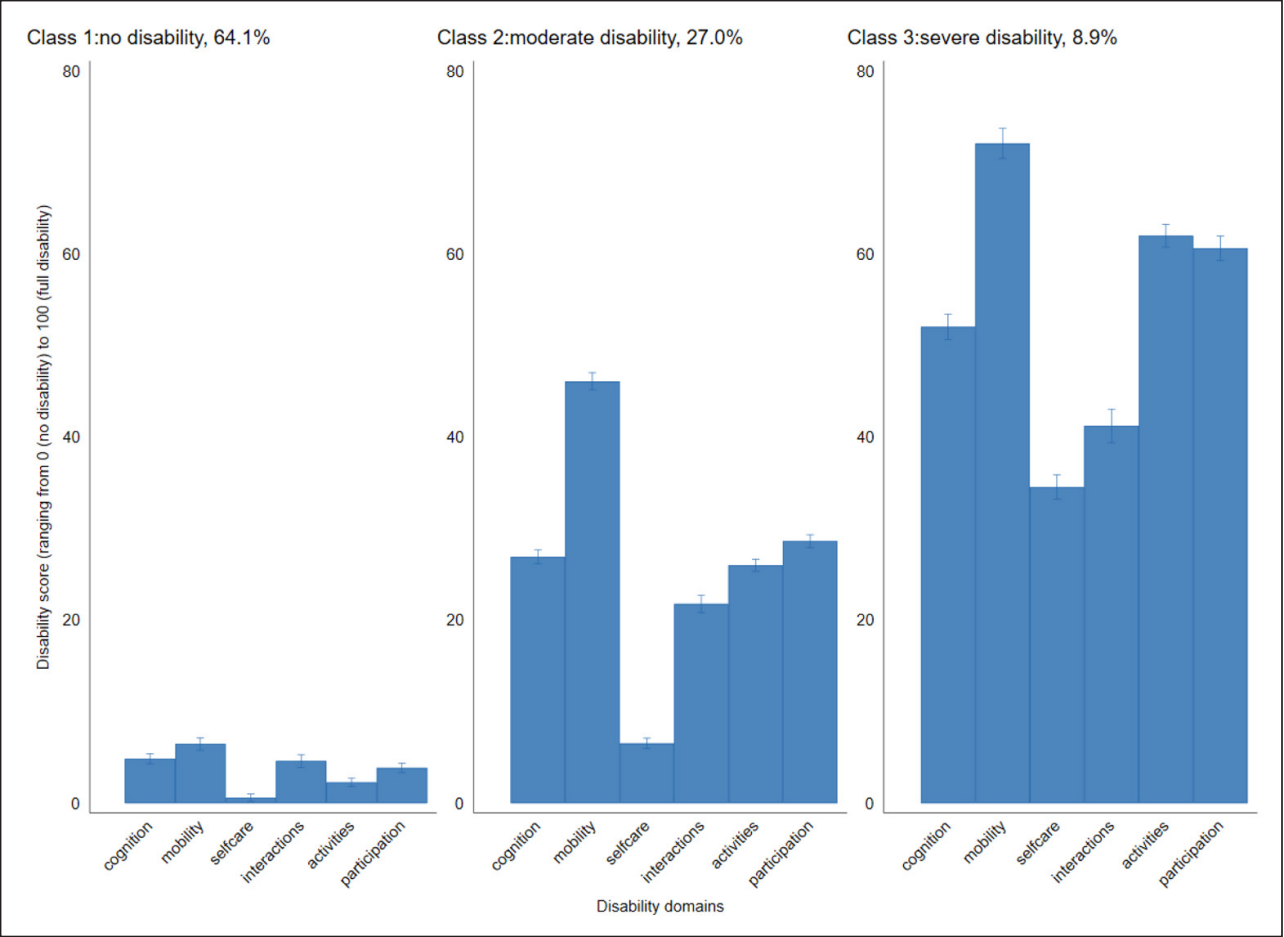
Latent classes of disability severity levels.

We ran LCA models from two to four classes selecting the three-class model based on indices of fit. The online supplementary file 3 shows the results of the fit indices. The three-class model had the lowest AIC and aBIC and thus was selected as the best fit model. Figure 2 shows the disability levels. Class one comprised participants with extremely low WHODAS scores (64.1%), thus labelled ‘no disability’. Participants with moderate WHODAS scores (27.0%) characterised class two and were thus labelled ‘moderate disability’, while those with high WHODAS scores (8.9%) characterised class three and thus labelled ‘severe disability’.

### Hierarchical cluster analysis findings

**Figure 3:**
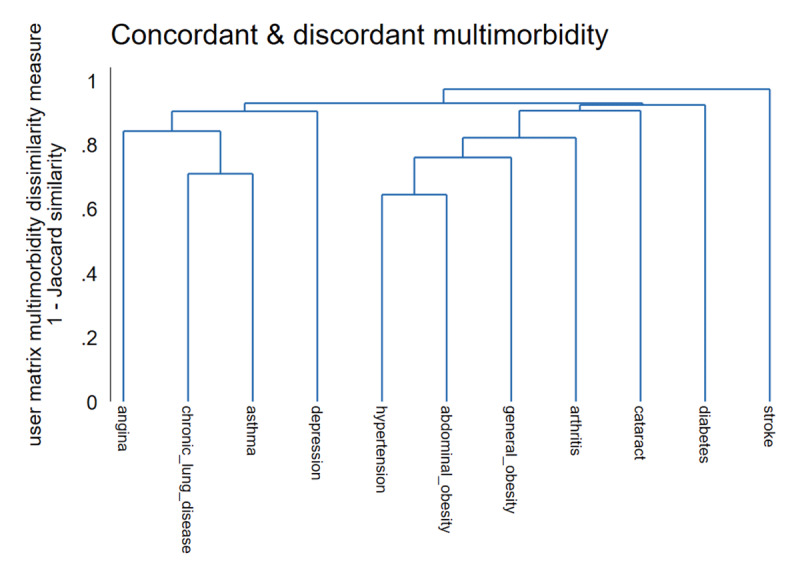
Dendrogram of concordant and discordant cardiometabolic multimorbidity clusters.

As a supplementary analysis, we also calculated multimorbidity patterns using the agglomerative hierarchical cluster analysis. Figure 3 shows the hierarchical tree plot of the multimorbidity clusters (dendrogram). The dendrogram shows a graphical representation of the agglomeration schedules at which multimorbidity clusters are combined. In general, the findings were similar to those obtained using LCA. The hierarchical clustering algorithms revealed distinct groupings of concordant and discordant cardiometabolic multimorbidity in the study sample. Based on the proximity coefficients, the first cluster comprised angina, chronic lung disease, asthma, and depression multimorbidity. The second cluster comprised hypertension, abdominal obesity, general obesity arthritis, cataracts, and diabetes multimorbidity.

### Distribution of disability severity levels

The distribution of disability severity levels is presented in Table 2. Severe disability was highest among older participants, females, unemployed, those with no formal education, and participants from South Africa. We found no difference in the severity levels of disability between rural and urban residents. Meanwhile, the prevalence of severe disability was highest among participants with discordant cardiometabolic multimorbidity comprising hypertension, abdominal obesity, diabetes, cataracts, and arthritis (23.5%), and those with angina, chronic lung disease, asthma, and depression multimorbidity (20.7).

**Table 2:** Distribution of disability severity levels.


CHARACTERISTICS	DISABILITY LEVELS		

	NO DISABILITY	MODERATE	SEVERE

	N = 2,387	N = 1,425	N = 378

Age, (mean) SD	59.6 (7.6)	63.9 (9.3)	68.4 (11.2)

** Sex			

Male	72.0	22.2	5.8

Female	57.6	30.9	11.5

**Education			

No formal education	51.0	36.0	13.0

Primary	62.6	27.8	9.5

Secondary	73.7	20.3	6.0

Tertiary	83.3	15.1	1.6

** Employment			

Public	70.3	26.1	3.5

Private	70.4	20.6	9.0

Self-employed	60.4	32.9	6.7

Informal employment	62.3	27.7	9.9

Informal	62.2	27.8	10.0

Unemployed	52.2	22.1	25.7

Residence			

Urban	64.5	26.0	9.5

Rural	63.7	29.1	7.3

*Study country			

Ghana	61.0	31.9	7.1

SA	66.3	23.7	10.0

**Concordant & discordant cardiometabolic multimorbidity classes			

Class 1: minimal cardiometabolic multimorbidity	68.0	26.2	5.9

Class 2: general and abdominal obesity class	66.6	25.5	7.9

Class 3: hypertension, abdominal obesity, diabetes, cataracts, and arthritis	44.0	32.5	23.5

Class 4: angina, chronic lung disease, asthma, & depression	47.5	31.8	20.7

Total	64.1	27.0	8.9


Cells are weighted row percentages, * p < 0.05, ** p < 0.001. *Class 1 comprised participants with minimal cardiometabolic multimorbidity. Class 2 comprised participants with high probabilities of general and abdominal obesity. Class 3 comprised participants with high probabilities of hypertension, diabetes, cataracts, and arthritis. Class 4 comprised participants with high probabilities of angina, chronic lung disease, asthma, and depression*.

### Cardiometabolic multimorbidity classes associated with moderate and severe disabilities

**Figure 4:**
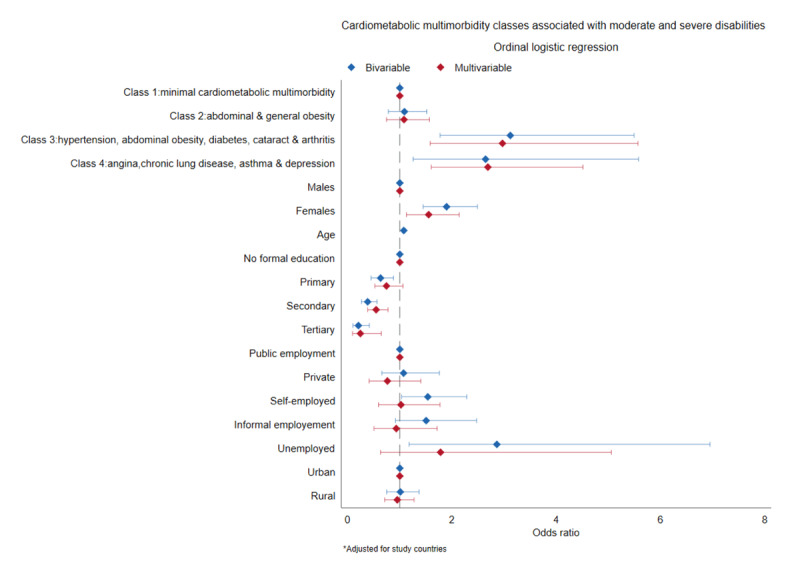
Ordinal regression of cardiometabolic multimorbidity classes associated with disability.

Figure 4 shows the results of the ordinal logistic regression model. Compared to the participants with minimal cardiometabolic multimorbidity, the odds of moderate and severe disabilities were higher among participants with discordant cardiometabolic multimorbidity comprising hypertension, abdominal obesity, diabetes, cataracts, and arthritis [aOR = 3.0; 95% CI 1.6 to 5.6], and those with angina, chronic lung disease, asthma, and depression [aOR = 2.7; 95% CI 1.6 to 4.5]. Being older was associated with higher odds of moderate and severe disabilities (aOR = 1.1; 95% CI 1.1 to 1.1). The odds of moderate and severe disabilities were lower among those with a secondary education (aOR = 0.5; 95% CI 0.4 to 0.8) and a tertiary education (aOR = 0.2; 95% CI 0.1 to 0.6) compared to those with no formal education.

## Discussion

In this study, we examined the latent classes of cardiometabolic multimorbidity associated with moderate and severe disability in Ghana and South Africa. We identified four distinct classes of cardiometabolic multimorbidity, including a relatively healthy group with minimal cardiometabolic multimorbidity: general and abdominal obesity; hypertension, abdominal obesity, diabetes, cataracts, and arthritis; and angina, chronic lung disease, asthma, and depression.

The cardiometabolic multimorbidity clusters identified in our study have similarities with findings from a systematic review of multimorbidity patterns in 14 studies drawn from across the world [53]. The review identified three broad patterns of multimorbidity comprising cardiometabolic, musculoskeletal, and mental health [53]. In our study, 10.0% of the participants were classified under the hypertension, abdominal obesity, diabetes, cataracts, and arthritis class, and 6.0% under the angina, chronic lung disease, asthma, and depression class.

Several underlying biological mechanisms could explain the clustering of concordant and discordant cardiometabolic multimorbidities identified in our study. Insulin resistance is well established in the literature as a possible pathophysiological mechanism explaining the clustering of CMDs [54,55,56]. Insulin resistance may affect the metabolism process and lead to abnormalities of vascular reactivity [6]. Lifestyle modifications to reduce metabolic syndrome and therapeutic intervention targeting insulin resistance may reduce the risk of cardiovascular diseases [57]. The clustering of angina, chronic lung disease, and asthma could be partly explained by inflammation, hypoxia, stress processes, and environmental risk factors such as smoking or air pollution [53,58].

Our results showed that 27.0% and 8.9% of the participants had moderate and severe disabilities with significant sociodemographic differences. Consistent with previous studies, being older, female, and having a low educational level were significantly associated with moderate and severe disabilities [59,60]. Although several previous studies have investigated the prevalence of disability among older adults, the heterogeneity in the definition of disability has hindered the comparison of findings. However, we selected three studies that are important for triangulation. The prevalence of ADL disability ranged from 1.6% to 16.6% in a study conducted in 2012 in South Africa and Ghana [61]. Another study evaluating the burden of disability using the SAGE Wave 1 survey found that 38.6% and 44.0% of older adults in South Africa and Ghana had disabilities [62]. Mitra et al. (2017) also estimated the global prevalence of disability to be 14% from a sample of 54 countries [32]. It is important to note that the disability severity levels were not investigated in the aforementioned studies. Moreover, only the physical components of disabilities were studied, while in the current study, we incorporated the diverse dimensions of disability comprising bodily level, person level, and societal level [20].

Relative to no disability, moderate and severe disabilities among older persons in Ghana and South Africa were significantly associated with two distinct discordant cardiometabolic multimorbidity classes comprising multimorbidity of hypertension, abdominal obesity, diabetes, cataracts, and arthritis and multimorbidity of angina, chronic lung disease, asthma, and depression. Although previous studies have also attributed disabilities to the presence of multimorbidity [1,21,22,23,24,25], the vast majority of literature is based on disease counts or indices with inadequate information on specific multimorbidity combinations [26,27]. Our findings add evidence to the discordant cardiometabolic multimorbidity combinations associated with moderate and severe disabilities to guide clinical and social interventions. However, there is still a lack of consensus on the pathway from the accumulation of chronic diseases to disabilities [63]. One possible explanation is the fact that multimorbidity may lead to anatomical and structural impairments, which results in functional limitations and finally, moderate and severe disabilities [64]. Further longitudinal studies are needed to determine the disability causal pathways and chronic disease aggregation.

### Strengths and limitations

This study has several strengths. First, data is from nationally representative population-based surveys of chronic conditions using a standardised WHO-SAGE protocol. Hence, the results are generalisable to the populations of the study countries. Second, the data used is based on direct measures of blood pressure, anthropometry, symptomatology algorithms, and self-reports, allowing for a more objective screening for chronic diseases than self-reporting used in over three-quarters of previous studies [26]. Third, the use of LCA in estimating the disability prevalence takes into consideration the heterogeneity of the disability severity levels frequently observed among the older population. Finally, the replication of the LCA results using the hierarchal cluster analysis of multimorbidity patterns strengthened the internal validity and robustness of the findings.

Our findings should be viewed while considering some limitations. First, the screening questions for disability and chronic diseases were partially based on self-reports. This may have resulted in the underestimation or overestimation of the true prevalence of disability severity levels and chronic diseases. However, previous studies in Ghana and South Africa have also reported consistent and similar prevalence rates [34,61,62]. Moreover, several other studies have shown reasonable validity and reliability between self-reported diagnoses and physician-diagnosed conditions [65,66].

Second, the number of CMDs and discordant chronic multimorbidity in the LCA was limited to those included in the SAGE survey. This may have left out other common chronic conditions among older persons, such as dementia and cancer, resulting in an underestimation of the multimorbidity prevalence. However, the observed prevalence of cardiometabolic multimorbidity in the current study is consistent with the findings of previous studies in Ghana and South Africa [67,68]. Future studies need to include more chronic diseases to increase external validity. Finally, the cross-sectional design of the data used in the current analysis implies that we cannot make conclusions regarding the temporality or causation between multimorbidity classes and disability. Future studies should use longitudinal analysis to estimate the incidence of transitions between latent classes and their impact on disability.

## Conclusions

Our results provide insight into the concordant and discordant cardiometabolic multimorbidity clusters associated with disability severity among older adults, using Ghana and South Africa as a case sample. Moderate and severe disabilities relative to no disability were associated with two distinct multimorbidity clusters comprising multimorbidity of hypertension, abdominal obesity, diabetes, cataracts, and arthritis and multimorbidity of angina, chronic lung disease, asthma, and depression. This evidence may be useful for defining disability prevention strategies and long-term care for older persons. Primary, secondary, and tertiary prevention of functional disability at the population and individual level should target older persons with or at risk of concordant and discordant cardiometabolic multimorbidities comprising hypertension, abdominal obesity, diabetes, cataracts, and arthritis and multimorbidity of angina, chronic lung disease, asthma, and depression.

## Data Accessibility Statement

Data used in the current study are publicly available on the microdata repository of the WHO (https://apps.who.int/healthinfo/systems/surveydata/index.php/catalog).

## Additional File

The additional file for this article can be found as follows:

10.5334/gh.1188.s1 Online supplementary Files. Files 1 to 3.

## Abbreviations

aBIC: Adjusted Bayesian Information Criterion

ADL: Activities of daily living

aOR: Adjusted odds ratio

CAIC: Akaike information criterion

CI: Confidence Interval

CMDs: Cardiometabolic diseases

DBP: Diastolic blood pressure

HCA: Hierarchical cluster analysis

LCA: Latent class analysis

SAGE: Survey of ageing and adult health

SBP: Systolic blood pressure

SSA: Sub-Saharan Africa

WHO: World Health Organization
