# Supplementary material for: Cardiometabolic Multimorbidity Associated with Moderate and Severe Disabilities: Results from the Study on Global AGEing and Adult Health (SAGE) Wave 2 in Ghana and South Africa
Source: Glob Heart. 2023 Mar 1;18(1):9. doi: 10.5334/gh.1188 (PMC9983501; doi:10.5334/gh.1188)
Supplement: Online supplementary Files. — Files 1 to 3. [file gh-18-1-1188-s1.pdf]

List of online supplementary files

Online supplementary file 1: Symptomatology algorithms

**Arthritis**	
Q1	During the last 12 months, have you experienced, pain, aching, stiffness, or swelling in or around the joints (like arms, hands, legs, or feet)which was not related to an injury and lasted for more than a month?
Q2	During the last 12 months, have you experienced stiffness in the joint in the morning after getting up from bed, or after a long rest of the joint without movement?
Q3	How long did this stiffness last?—1) less than 30 minutes; 2) more than 30 minutes `
Q4	Did this stiffness go away after exercise or movement in the joint?—1) yes; 2) no
Algorithm<br>If the response to Q1 and 2 was "yes" and the response to questions 3 and 4 was the first option, the respondent was said to have arthritis	
**Angina**	
Q1	During the last 12 months, have you experienced any pain or discomfort in your chest when you walk uphill or hurry?
Q2	During the last 12 months, have you experienced any pain or discomfort in your chest when you walk at an ordinary pace on level ground?
Q3	What do you do if you get the pain or discomfort when you are walking?—1) stop or slow down; 2) carry on after taking a pain-relieving medicine that dissolves in your mouth; 3) carry on walking
Q4	If you stand still, what happens to the pain or discomfort?—1) relieved; 2) not relieved
Q5	Apart from these questions, respondents were asked to identify the points of pain in the upper part of the body (excluding the head) with the help of a picture depicting the upper parts of the body.
Algorithm<br>If the response to Q1 and Q2 was "yes" and the response to Q3 and 4 was the first option, and in Q5 the respondent indicated that the pain was in the upper left part of the body, the person was said to have angina.	
**Chronic Lung Disease**	
Q1	During the last 12 months, have you experienced any shortness of breath at rest (while awake)?
Q2	During the last 12 months, have you experienced any coughing or wheezing for 10 minutes or more at a time?
Q3	During the last 12 months, have you experienced any coughing up of sputum or phlegm on most days of the month for at least 3 months?
Algorithm<br>A respondent was ascertained to have chronic lung disease if his/her response was "yes" to Q1 or "yes" to both Q2 and Q3.	
**Asthma**	
Q1	During the last 12 months, have you experienced attacks of wheezing or whistling breathing?
Q2	During the last 12 months, have you experienced an attack of wheezing that came on after you stopped exercising or some other physical activity?
Q3	During the last 12 months, have you had a feeling of tightness in your chest?
Q4	During the last 12 months, have you woken up with a feeling of tightness in your chest in the morning or any other time?
Q5	During the last 12 months, have you had an attack of shortness of breath that came on without an obvious cause when you were not exercising or doing some physical activity?
Algorithm<br>A respondent was said to suffer from asthma if s/he responded "yes" to Q1 and "yes" to any of the subsequent Q2–Q5.	

**Online supplementary file 1 continued**	
**Depression**	
Q1	During the last 12 months, have you had a period lasting several days when you felt sad, empty, or depressed?
Q2	During the last 12 months, have you had a period lasting several days when you lost interest in most things you usually enjoy, such as personal relationships, work, or hobbies/recreation?
Q3	During the last 12 months, have you had a period lasting several days when you have been feeling your energy decreased or that you are tired all the time?
If the response to any of the above 3 questions was "yes," then the following set of questions was asked:	
Q4	Did this period (of sadness/loss of interest/low energy) last for more than 2 weeks?
Q5	Was this period (of sadness/loss of interest/low energy) most of the day, nearly every day?
Q6	During this period, did you lose your appetite?
Q7	Did you notice any slowing down in your thinking?
Q8	Did you notice any problems falling asleep?
Q9	Did you notice any problems waking up too early?
Q10	During this period, did you have any difficulties concentrating—for example, listening to others, working, watching television, listening to the radio?
Q11	Did you notice any slowing down in your moving around?
Q12	During this period, did you feel anxious and worried most days?
Q13	During this period, were you so restless or jittery nearly every day that you paced up and down and could not sit still?

	Online supplementary file 1 continued
Q14	During this period, did you feel negative about yourself or like you had lost confidence?
Q15	Did you frequently feel hopeless—that there was no way to improve things?
Q16	During this period, did your interest in sex decrease?
Q17	Did you think of death, or wish you were dead?
Q18	During this period, did you ever try to end your life?

Algorithm

To ascertain depression from this set of questions, 2 sets of variables were computed. The first set of variables was based on Q1–Q5 and Q16. From this set, 3 variables were computed taking the values 0 and 1, as follows:

1. The first variable takes the value 1 if the response to any of Q1, Q4, and Q5 is "yes."
2. The second variable takes the value 1 if the response to Q2 or Q16 is "yes."
3. The third variable takes the value 1 if the response to Q3 is "yes."

The second set of variables was based on Q6–Q15, Q17, and Q18. From these questions, 7 variables were computed.

1. The first variable takes the value 1 if the response to Q14 or Q15 is "yes."
2. The second variable takes the value 1 if the response to Q12 or Q13 is "yes."
3. The third variable takes the value 1 if the response to Q17 or Q18 is "yes."
4. The fourth variable takes the value 1 if the response to Q7 or Q10 is "yes."
5. The fifth variable takes the value 1 if the response to Q11 is "yes."
6. The sixth variable takes the value 1 if the response to Q8 or Q9 is "yes."
7. The seventh variable takes the value 1 if the response to Q6 is "yes."

These newly created variables from the respective sets were added to obtain 2 new variables, the first consisting of the sum of the first set of variables (maximum value 3) and the second consisting of the sum of the second set of variables (maximum value 7). Based on these 2 variables, a respondent was said to suffer from depression if s/he had a value for the first variable of 2 or more and a value for the second variable of 4 or more

Online supplementary file 2: A comparison of the characteristics of complete cases and incomplete cases

Background Characteristics	Complete cases	Incomplete cases	Std. Diff
Number of study participants	4,190	1,567	
Proportion (%)	72.8%	27.2%	
Mean age: All participants (SD)	64.1 (10.0)	65.8 (11.0)	0.16
Males (SD)	64.9 (10.1)	66.9 (10.9)	0.19
Females (SD)	63.5 (9.9)	65.3 (11.0)	0.17

SD: standard deviation, Std Diff: standard difference. Std Diff= Difference in means divided by standard error; imbalance defined as an absolute value greater than 0.2

Online supplementary file 3; Comparison between latent class models

Number of latent classes		CAIC	aBIC
Concordant & discordant cardiometabolic comorbidities			
	2	32427.4	32566.9
	3	31934.4	32156.3
	4	**31791.5**	**32089.5**
	5	31748.2	32103.3
Disability (WHODAS scores)			
	2	212065.2	212185.6
	3	**206668.7**	**206833.5**
	4	206682.7	206891.9

Note: Boldface type indicates the selected model. aBIC adjusted Bayesian Information Criterion, CAIC consistent Akaike Information Criterion, WHODAS, World Health Organization Disability Assessment
